# Recruitment into trials of rare conditions - experiences from the STOP GAP trial

**DOI:** 10.1186/1745-6215-12-S1-A109

**Published:** 2011-12-13

**Affiliations:** 1 University of Nottingham, Nottingham, NG7 2NR, UK

## Objective

To highlight a successful method for improving recruitment into a trial of a rare skin condition.

## Background

Pyoderma gangrenosum (PG) is a rare, ulcerative condition that is often associated with underlying autoimmune disease. Most dermatologists in the UK only see 1-2 PG patients per annum, making this a very difficult condition to evaluate empirically.

## Methods

The STOP GAP trial is a randomised controlled trial (RCT) of oral prednisolone compared to oral ciclosporin for the treatment of PG. The study includes a parallel observation study (case series) for patients requiring topical therapy.

Patients who are enrolled into the observational study continue to be followed up and contribute data relating to the efficacy and acceptability of topical treatments. These data provide an important contribution to the available literature on PG, which to-date is based largely on case reports and retrospective case series. An additional benefit of this approach is that patients remain in contact with the research team, and should systemic therapy be indicated, participants are considered for inclusion into the RCT of systemic treatments. This design is efficient and means that most patients who are willing and able to give informed consent are able to contribute to the research activity; this contributes to a broader evidence-base for the treatment of PG patients.

## Results

The STOP GAP trial is ongoing and has currently enrolled 73/140 (52%) participants into the RCT, and 35 participants into the observational study. Five (14%) of the patients given topical therapy (observational study) have subsequently gone on to take part in the RCT, having required systemic treatment.

## Conclusions

Whilst it is more work to conduct an observational study alongside an ongoing RCT, and the added value of these data is limited by the lack of a randomised comparator, the benefits of this approach outweigh the disadvantages when the condition of interest is rare, and where the existing evidence base is poor.

Trial registration: ISRCTN 35898459

